# Human esophageal myofibroblasts increase squamous epithelial thickness via paracrine mechanisms in an in vitro model of gastroesophageal reflux disease

**DOI:** 10.1371/journal.pone.0238852

**Published:** 2020-09-14

**Authors:** Liping Hu, Chunying Zhang, Kevin Yang, Meng Li, Anisa Shaker

**Affiliations:** 1 Department of Medicine, Division of Gastroenterology and Hepatology, Keck School of Medicine of USC, Los Angeles, California, United States of America; 2 USC Libraries Bioinformatics Services, University of Southern California, Los Angeles, California, United States of America; Peter MacCallum Cancer Centre, AUSTRALIA

## Abstract

The pathogenesis of esophageal injury in gastroesophageal reflux disease (GERD) is incompletely understood. We modeled exposure of human esophageal myofibroblasts (HEMFs) to gastroesophageal reflux by repeated treatment with pH 4.5 and pH 4.5 bile salts and determined the effects on the epithelium in a 3D organotypic-like air-liquid interface model. Total, basal and supra-basal thickness of the epithelium were measured and immunostaining for p63, for basal (CK 14) and supra-basal (CK 4) squamous differentiation markers, and for cell proliferation (PCNA) were performed. Epithelial cell proliferation in response to HEMF conditioned media was also assessed in 2D culture. In the 3D organotypic model, total epithelial thickness increased similarly with pH 4.5 and pH 4.5 bile salt treated versus untreated and bile salt treated HEMF conditioned media. Epithelial p63 immunostaining was increased and multilayered. There was expansion of the CK14+ basal and CK4+ supra-basal layers in the epithelium established with conditioned media from pH 4.5 and pH 4.5 bile salt treated HEMFs versus untreated HEMF conditioned media. PCNA + cells per μm of tissue were unchanged in the basal layer across all treatment conditions while PCNA + cells per total DAPI + cells were decreased. In 2D culture, basal epithelial proliferation decreased with conditioned media from pH 4.5 and pH 4.5 bile salt treated HEMFs compared to conditioned media from untreated HEMF conditioned media. Secreted factors from HEMFs treated with acidic stimuli encountered in GERD increase epithelial thickness compared to secreted factors from untreated HEMFs and expand both basal and supra-basal layers. Our findings demonstrate for the first time paracrine regulation of the squamous epithelium from acid stimulated HEMFs. The effects of secreted factors from acid treated HEMFs on basal cell proliferation in this model and the mechanism mediating the increase in epithelial thickness merit further investigation.

## Introduction

Squamous epithelium in non-erosive and erosive gastro-esophageal reflux disease (GERD) is characterized by an increase in epithelial thickness and dilated intercellular spaces [1, 2]. An increase in T lymphocytes is also associated with reflux esophagitis [3]. The mechanisms mediating these histologic changes and the pathogenesis of reflux esophagitis continue to be an active area of investigation. Noxious luminal factors implicated in GERD pathogenesis include an acidic refluxate such as acidic bile salts [4]. Work in animal models [5], recently confirmed in human tissue [3], suggests the mechanism of injury in GERD is not a direct caustic effect of acid but rather an inflammatory response that begins in the sub-epithelium.

Interestingly, the effect of noxious luminal factors on the sub-epithelial non-immune cellular components has not been rigorously investigated. We previously described human esophageal myofibroblasts (HEMFs) subjacent to the basal layer of the squamous epithelium in normal human esophagus [6]. We observed an increase in this population in de-identified biopsies from patients with histology typical of GERD (basal intracellular edema, intraepithelial squamous infiltration by neutrophils, lymphocytes and eosinophils, basal cell hyperplasia, and elongation of vascular papillae) as well as NF-ĸB activation in sub-epithelial HEMFs [6]. Cytokine arrays performed on conditioned media collected from primary cultures of HEMFs established from normal human esophagi as well as a HEMF cell line [7] established in our lab demonstrate that HEMFs respond to one time treatment with pH 4.5 and TLR4 ligands with secretion of pro-inflammatory cytokines, predominantly IL8 and IL6 [6]. We have also previously shown that growth factors in conditioned media from untreated HEMFs increase epithelial proliferation and thickness in a 3D organotypic-like air-liquid interface model (3D OTC-ALI), with expansion limited to the basal layer [8].

Our hypothesis therefore was that stimulation of HEMFs with noxious luminal factors such acid and acidic bile salts encountered in GERD would result in secretion of factors that also impact overlying epithelial cells. We hypothesized that secreted factors from stimulated HEMFs would regulate epithelial basal cell proliferation (hyperplasia) and epithelial thickness. In this study, we aimed to model GERD exposure of HEMFs by treatment with acid or acidic bile salts and determine the effects of treated HEMF conditioned media on squamous epithelial cells in 2D and 3D organotypic models.

## Materials and methods

### Cell lines and culture

A previously characterized and validated human esophageal myofibroblast (HEMF) cell line [7], herein referred to as HEMFs, was cultured in myofibroblast media as previously described [6, 7]. This protocol was approved by the Institutional Review Board of Keck School of Medicine of University of Southern California and deemed coded specimens/data. Myofibroblast media consists of DMEM with 10%FBS, 14.8ug/ml insulin (Sigma. Cat# I9278), 20.4 μg/ml transferrin (Sigma cat# T8158), 0.1mg/ml gentamicin (Sigma. Cat#G1397), and 8ng/ml EGF. For experiments requiring collection of HEMF conditioned media, HEMFs were grown in EGF-free media in the peri-study period to mitigate the effect of EGF normally present in HEMF media on epithelial cells. HEMF cell lines were treated for 8 min every 2 hr x 4 with the following possible treatments: 1. serum-free, EGF-free myofibroblast media (SFMM); 2. SFMM acidified to pH 4.5 with hydrochloric acid; 3. SFMM with bile salt mixture (glycocholic, taurocholic, glycochenodeoxycholate, and taurochenodeoxycholic-, glycodeoxycholic-, and taurodeoxycholic-acids) in a 20:3:15:3:6:1 molar concentration as per [9]; or 4. pH 4.5 bile salt in SFMM (bile salt mixture in SFMM acidified to pH 4.5). In between treatments, HEMFs were grown in full myofibroblast media. After the last treatment, HEMFs were washed with PBS three times and then incubated overnight in SFMM. The next morning, cells were harvested and supernatant (conditioned media) was collected for analysis. A human esophageal epithelial cell line EPC-hTERT (a human telomerase reverse transcriptase-immortalized esophageal cell line that was a kind gift from Dr. Anil Rustgi) were cultured as previously described [7, 8] in KSFM or in 3D OTC-ALI as described below.

#### Squamous epithelial cell proliferation assays

Proliferation of an immortalized epithelial cell line in 2D culture treated with HEMF supernatants was determined with the alarmarBlue cell viability assay (cat# DAL1025, ThermoFisher) and Celltiter-Glo (CTG) luminescent cell viability (Promega) assays according to manufacturer’s instructions. Briefly, STR cells were placed in a 96 well plate (600 cells/well) and incubated at 37° Celsius, 5% CO_2._ STR were then incubated with supernatants collected from untreated, pH 4.5 and pH 4.5 bile salt treated HEMFs grown in serum free, EGF free media at 1:1 (neat), 1:4, and 1:8 dilutions with SFMM for 72 or 96 hr. Serum-free, EGF free myofibroblast media (SFMM) and keratinocyte serum-free media (KSFM) without dilutions were used as controls for each corresponding condition which was diluted. Dilutions for supernatant/conditioned media from treated HEMFs were performed with SFMM. Alamar Blue (10μl/well) was then added, incubated at37° Celsius for 5 hrs, and fluorescence recorded using Plate Reader. For CTG assay, several pilot studies were conducted to define optimum treatment conditions. STR cells were placed in 96 well plate (2000 cells/well). Controls were KSFM and SFMM diluted with 1:1 with KSFM. Supernatants from untreated and treated HEMFs (cSFMM, pH 4.5 cSFMM, and pH 4.5 bile salt cSFMM) were also diluted 1:1 with KSFM. A volume of CellTiter-Glo^®^ Reagent equal to the final volume of cell culture medium present in each well (50 μl) was added. Samples were mixed and incubated at room temperature for 11 minutes, then luminescence was recorded with Plate Reader. Epithelial cells were not treated directly with acidified media or acidified media bile salt mixture, only with conditioned media from untreated or treated HEMFs, described above.

### 3D OTC-ALI

A 3D organotypic-like air-liquid interface model that phenocopies stratified squamous epithelium (3D OTC-like ALI) wherein upper chamber epithelial cells are co-cultured with lower chamber untreated control (cSFMM) or conditioned media from pH4.5 or pH4.5 bile salt treated HEMFs was used to determine the effect of secreted factors from HEMFs on the epithelium as previously described [8]. All conditions were studied in triplicate at least three times.

Briefly, STR cells were grown to confluence in low Ca2^+^ keratinocyte serum free media (0.09 mM calcium). The medium was changed to high Ca2^+^ by the addition of extracellular calcium (final concentration 1.8 mM) for days 3–7. Media was refreshed on day 5. Then on day 8, air-liquid interface was induced by removing the media from the upper chamber. Conditioned media was added to the lower chamber and refreshed every other day until day of harvest (day 14). Cultures were fixed with 10% neutralized buffered formaldehyde in the plate at 4° for 1 hour and submitted for histology.

### Immunohistochemistry and immunofluorescence

We performed immunohistochemistry and immunofluorescence on paraffin-embedded, formalin-fixed sections of 3D organotypic ALI cultures as previously described [8]. 5 μm sections were stained with hematoxylin-eosin and evaluated under a Nikon microscope with 40x objective. Total epithelial thickness was measured as previously described [8]. Briefly, to quantify epithelial thickness, we examined the entire length of each available strip (at least 3 strips) on each slide with a 40x objective. For each field of vision (FOV), total epithelial thickness was measured at each end of the visible strip of tissue as well as in the middle, for a total of 3 at least measurements. Primary antibodies used for immunofluorescence were rabbit polyclonal p63 (1:500, Genetex), mouse anti-PCNA (1:200, BD Transduction, cat#610664), mouse anti-CK14 (1:600, Abcam, ab7800), rabbit anti-CK4 (1:100, Abcam, ab51599), followed by goat anti-rabbit CY2 (1:1000), goat anti-mouse CY5 (1:600), or rodamine goat anti-mouse (1:200) secondary antibodies (Jackson ImmunoResearch). Immunofluorescent slides were examined with a confocal microscope (Leica TCS SP8-Leica microsystems) at 40x. To quantify the number of p63 and PCNA positive cells per um of tissue, p63+ and PCNA+ positive cells were counted and normalized to the length of the epithelial strip visible and to the total number of DAPI + cells in each FOV at 40x. Basal and supra-basal thickness were quantified by measuring thickness of CK14 (basal differentiation marker) and CK4 (supra-basal differentiation marker) co-immunostained strips of tissue, for each condition, as described above.

### Statistics/analysis

All experiments were performed at least in triplicate and data presented as means ± SE or as box and whisker plot of data (minimum, maximum, lower and upper quartile, and median are shown in the box and whisker plot). Mean values are indicated by X. Data were analyzed using Student’s two-tailed type 2 t-test or ANOVA with a Tukey’s or Dunnett’s post hoc test, as appropriate with GraphPad Prism 6.0 (GraphPad Software, La Jolla, CA). A value of P < 0.05 indicated statistical significance. Materials, data, and associated protocols are available upon request. All authors had access to the study data and had reviewed and approved the final manuscript.

## Results

### Conditioned media from acid and acidic bile salt treated HEMFs increases squamous epithelial thickness in 3D OTC-ALI

**Fig 1:**
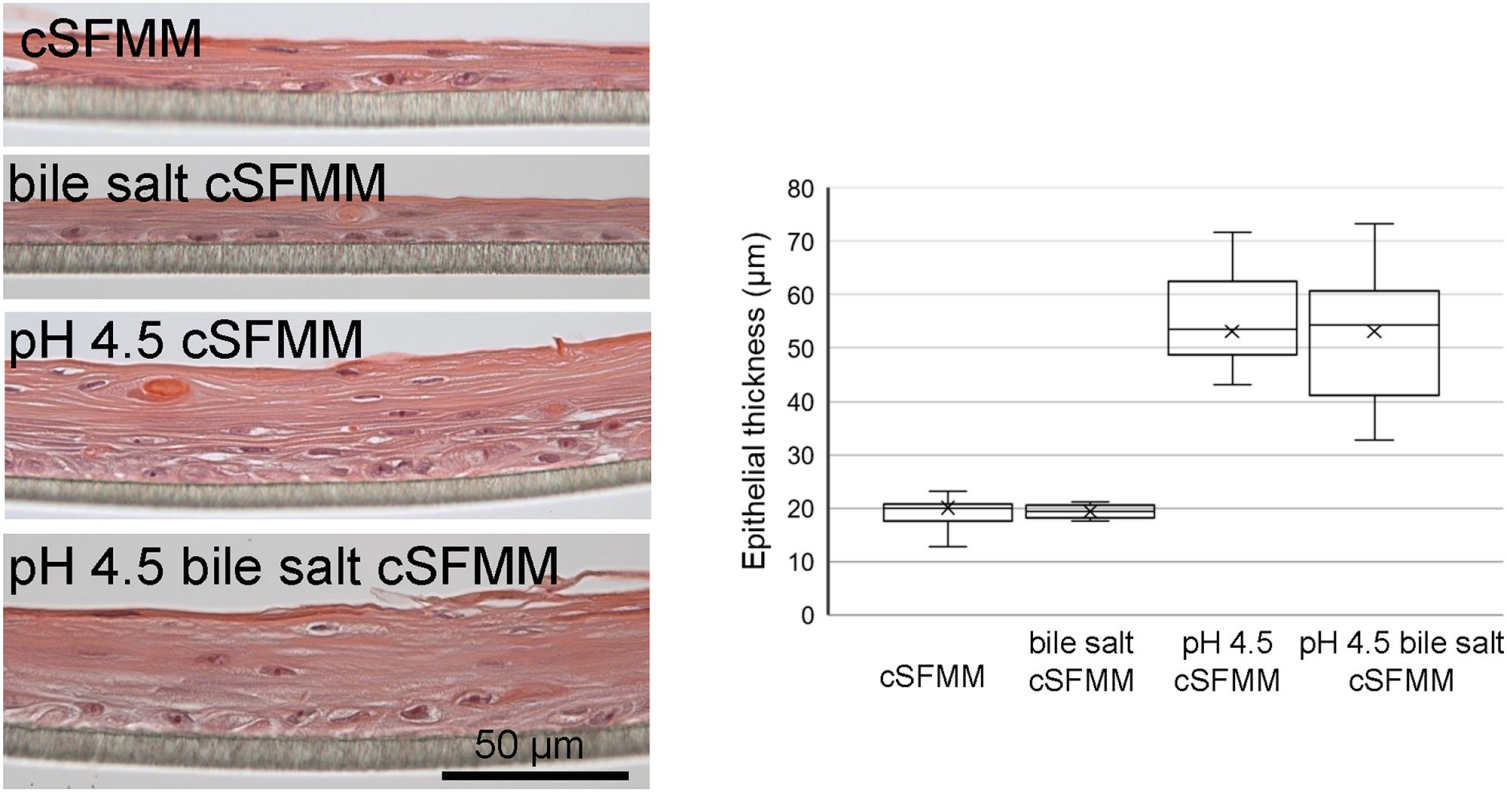
Effect of conditioned media from pH 4.5 and pH 4.5 bile salt treated HEMFs on the esophageal squamous epithelium in 3D OTC-ALI. pH4.5 bile salt treated HEMF conditioned media increases total epithelial thickness vs untreated serum-free, EGF-free HEMF conditioned media (cSFMM). (A). Representative images of three independent, reproducible experiments are shown. Scale bar represents 50 μm. (B). Box and whisker plot of data from three independent experiments is shown (minimum, maximum, lower and upper quartile, and median are shown in the box and whisker plot). Mean values are indicated by X. Total epithelial thickness is increased in the squamous epithelium established with conditioned media from pH 4.5 or vs pH 4.5 bile salt treated HEMFs vs conditioned media from untreated HEMFs or HEMFs treated with bile salts alone (mean values 53.0 μm vs 53.1 μm vs 20.0 μm and 19.4 μm, p<0.005 for cSFMM vs pH 4.5 cSFMM and pH 4.5 bile salt cSFMM; p<0.005 for bile salt cSFMM vs pH 4.5 cSFMM and pH 4.5 bile salt cSFMM).

The lining of the human esophagus is a stratified squamous epithelium. Biopsies from GERD patients have shown an increase in total epithelial thickness [2]. We therefore wanted to evaluate the effect of HEMF conditioned media on epithelial proliferation and differentiation in a model most reflective of esophageal physiology. As such we utilized the well-established 3D organotypic-ALI model (3D OTC-ALI) [8]. Total epithelial thickness increases in 3D OTC -ALI established with conditioned media from pH 4.5 and pH 4.5 bile salt treated HEMFs compared to untreated HEMF conditioned media or HEMFs treated with bile salts alone (mean thickness 53.0 μm and 53.1 μm vs 20.0 μm and 19.4 μm vs, p<0.005) (Fig 1). Because we did not observe an effect with conditioned media from bile salt treated HEMFs, additional studies were not performed with this condition.

**Fig 2:**
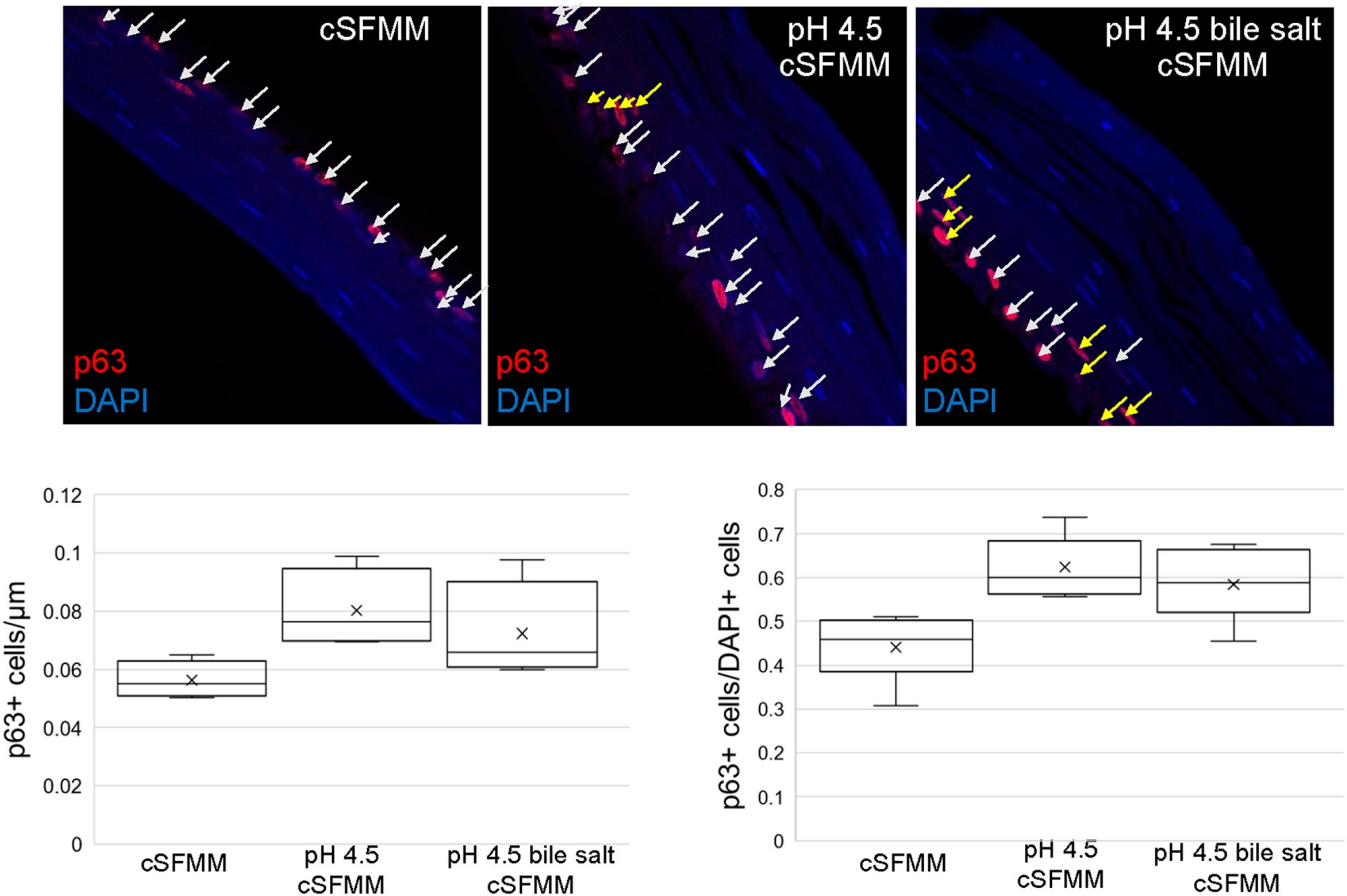
Basal cell marker p63 expression in the squamous epithelium in 3D OTC-ALI in response to conditioned media from untreated, pH 4.5 and pH4.5 bile salt treated HEMFs. p63 (pink/red) immunostaining is shown for the epithelium from 3D-OTC established with conditioned media from untreated, pH 4.5, and pH 4.5 bile salt treated HEMFs. p63 signal is multi-layered in the squamous epithelium established with conditioned media from pH 4.5 and pH 4.5 bile acid treated HEMFs. Arrows point to p63 stained cells and yellow arrows indicate multi-layered cells. Representative images and quantification of three independent, reproducible experiments are shown. Quantification of p63 was done per total length (μm) of tissue available for histologic analysis and also per DAPI+ cells. Box and whisker plot of data from three independent experiments is shown (minimum, maximum, lower and upper quartile, and median are shown in the box and whisker plot). Mean values are indicated by X. An increase in p63+ cells/μm is observed in the epithelium established with conditioned media from pH 4.5 treated HEMFs (separate box and median) and a trend in increase expression with conditioned media from pH 4.5 bile salt treated HEMFs compared to untreated HEMFs (cSFMM and pH 4.5 bile salt conditioned media boxes overlap, but median of pH 4.5 bile salt conditioned media remains outside of cSFMM box). When p63 quantification is performed per DAPI+ cells, an increase in the number of p63+ cells is observed in the squamous epithelium established with conditioned media from pH 4.5 and from pH 4.5 bile salt treated HEMFs compared to conditioned media untreated HEMFs.

To begin to define the mechanisms underlying the observed increase in thickness, we performed immunostaining for p63, a transcription factor expressed by basal cells [10] (Fig 2). p63 immunostaining of squamous epithelium established with pH 4.5 and pH 4.5 bile salt treated HEMF conditioned media was multi-layered, while immunostaining in epithelium established with untreated HEMF conditioned media was a single layer. An increase in p63+ cells per μm of tissue is observed in epithelium established with conditioned media from pH 4.5 treated HEMFs and a trend in increase p63 signal with conditioned media from pH 4.5 bile salt treated HEMFs compared to untreated HEMFs. When quantified per DAPI+ cells, an increase in p63+ cells is observed in squamous epithelium established with conditioned media from pH 4.5 treated HEMFs and from pH 4.5 bile salt treated HEMFs compared to untreated HEMFs.

**Fig 3:**
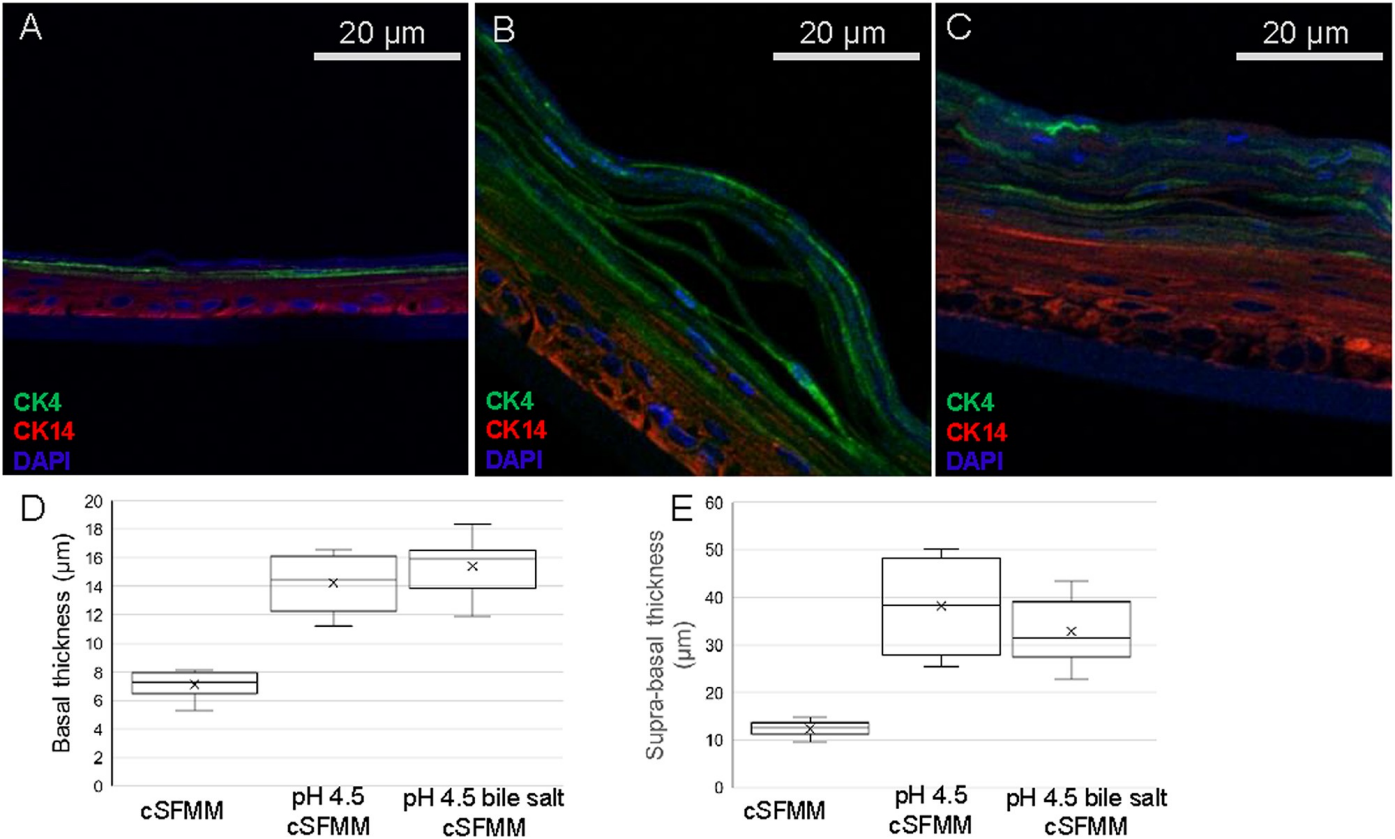
Immunofluorescence for basal and supra-basal differentiation markers and basal/supra-basal thickness of the squamous epithelium in 3D OTC-ALI established with conditioned media from untreated, pH 4.5, and pH 4.5 bile salt treated HEMFs. Basal (red, CK 14) and supra-basal (green, CK 4) immunofluorescent staining shown in panels A-C for the epithelium in 3D OTC established with conditioned media from untreated (A), pH 4.5 treated (B), and pH 4.5 bile salt treated (C) HEMFs. Overlap between basal (red, CK 14) and supra-basal (green, CK 14) differentiation markers is visible in the epithelium established with conditioned media from pH 4.5 (B) treated HEMFs. Representative images of three independent, reproducible experiments are shown. (D and E) Box and whisker plot of data from three independent experiments is shown (minimum, maximum, lower and upper quartile, and median are shown in the box and whisker plot). Mean values are indicated by X. (D) Quantification of basal epithelial thickness as defined by CK14 immunostaining shows an increase in mean basal thickness of the epithelium established with conditioned media from pH 4.5 and pH 4.5 bile salt treated HEMFs (mean values: 7.1 μm vs. 14.2 μm and 15.4 μm, p< 0.05; ranges of CK14 + cell layer thickness in cSFMM, pH 4.5 cSFMM, and pH 4.5 bile salt samples are 6.6–8.0 μm; 11.2–16.5 μm; and 11.8–18.4 μm, respectively) (E) Quantification of supra-basal thickness as defined by CK4 immunostaining shows an increase in mean supra-basal thickness of the epithelium established with conditioned media from pH 4.5 and pH 4.5 bile salt treated HEMFs (mean values: 12.3 μm vs 38.1 μm and 32.8 μm, p< 0.05; ranges of CK4 + cell layer thickness in cSFMM, pH 4.5 cSFMM, and pH 4.5 bile salt samples are 9.5–13.8 μm; 25.5–50.1 μm; and 22.7–43.5 μm).

We then performed immunostaining for basal and supra-basal differentiation markers with immuno-staining with cytokeratin filaments (CKs) which are major constituents of esophageal epithelium (Fig 3). As we had observed in H&E stained sections, in sections obtained for immunofluorescence, the thickness of the squamous epithelium established with pH 4.5 and pH 4.5 bile salt treated HEMF conditioned media remained noticeably thicker. We proceeded to perform co-immunostaining for CK14 which is a basal differentiation marker and for CK4 which is a supra-basal differentiation marker. We then quantified basal and supra-basal epithelial thickness based on respective immunostaining. Basal thickness was increased in the epithelium established with conditioned media from pH 4.5 and pH 4.5 bile salt treated HEMFs compared to untreated HEMFs (14.2 μm and 15.4 μm vs 7.1 μm, p< 0.05). Similarly, there was an increase in supra-basal thickness in epithelium established with conditioned media from pH 4.5 and pH 4.5 bile salt treated HEMF conditioned media compared to untreated HEMFs (38.1 μm and 32.8 μm vs 12.3 μm, p < 0.05).

### The effect of acid and acidic bile salt treated HEMF conditioned media on squamous cell epithelial proliferation

**Fig 4:**
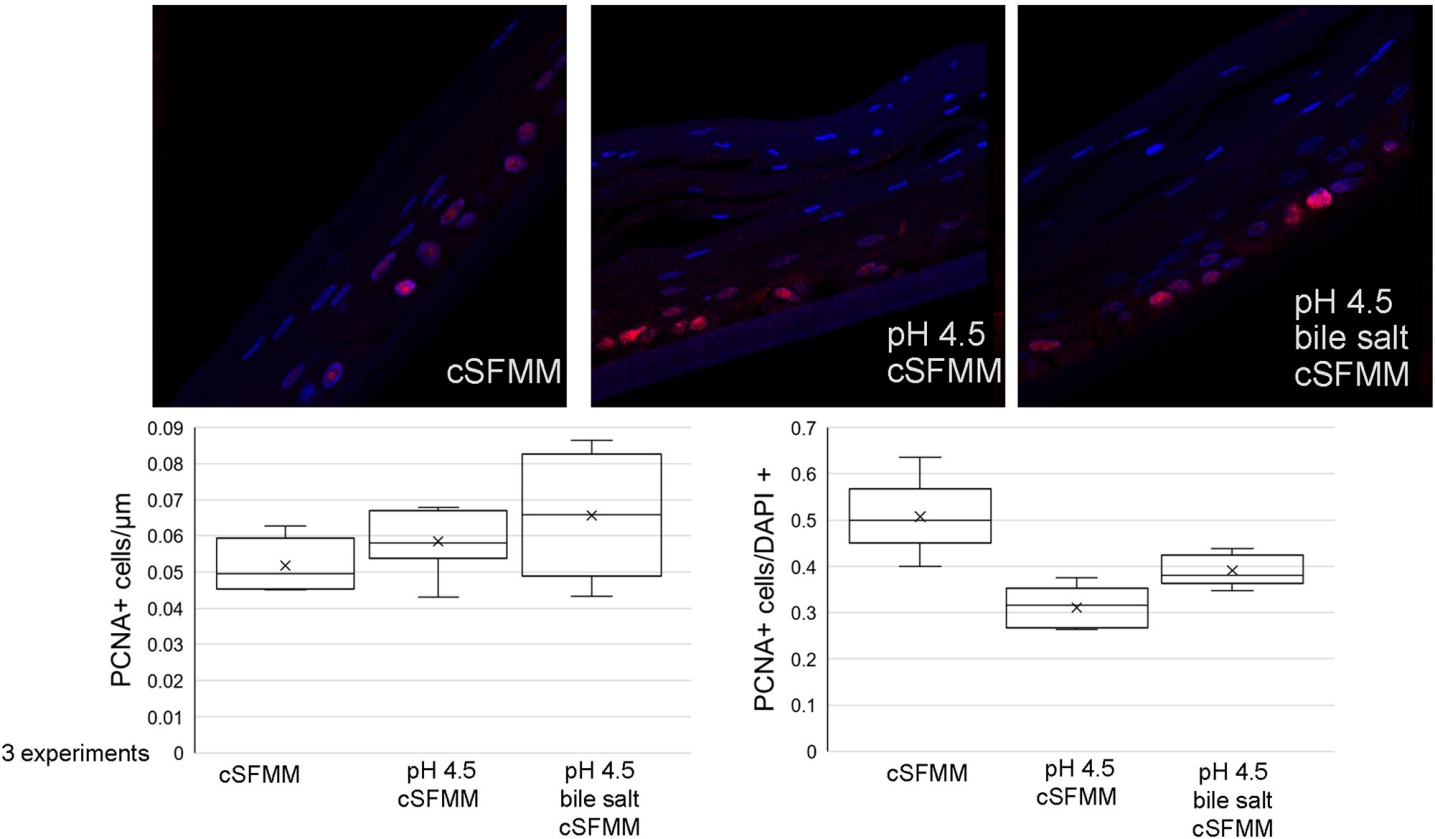
Proliferative marker PCNA expression in the squamous epithelium in 3D OTC-ALI in response to conditioned media from untreated, pH4.5 and pH4.5 bile salt treated HEMFs conditioned media. PCNA immunostaining (pink/red) is limited to the lowest layer cells in the squamous epithelium established with conditioned media from untreated, pH4.5 treated, and pH4.5 bile salt treated HEMFs. Quantification of PCNA + cells per μm of tissue length and per DAPI+ cells are shown in the graphs. Quantification of PCNA per μm of tissue length is similar across all conditions. Quantification of PCNA per DAPI+ cells demonstrates a decrease in PCNA expression in epithelial cells established with conditioned media from pH 4.5 and pH 4.5 bile salt treated HEMFs vs untreated HEMFs. Box and whisker plot of data from three independent experiments is shown (minimum, maximum, lower and upper quartile, and median are shown in the box and whisker plot). Mean values are indicated by X.

To determine if the increase in epithelial thickness was due to an increase in epithelial cell proliferation, we proceeded with immunostaining for proliferating cell nuclear antigen (PCNA) (Fig 4). PCNA immunostaining was evident in the lower epithelial layers in all treatment conditions. We quantified PCNA normalized to length of tissue examined as well as to number of DAPI+ cells. Quantification of PCNA per μm of tissue did not demonstrate s difference in PCNA expression amongst the three treatment conditions. However, quantification of the number PCNA+ cells per DAPI+ cells showed a decrease in PCNA expression in squamous epithelium established with conditioned media from pH 4.5 and pH 4.5 bile salt treated HEMFs compared to untreated HEMF conditioned media.

**Fig 5:**
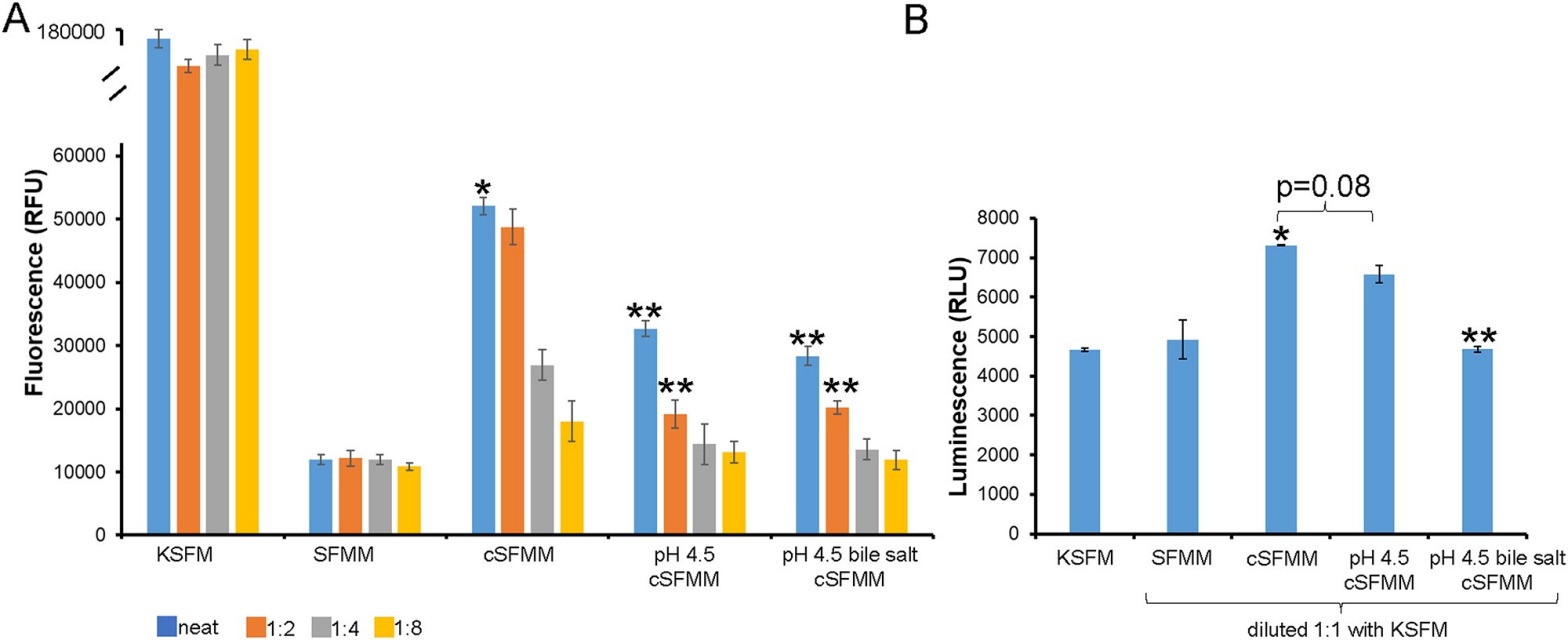
Esophageal epithelial basal cell proliferation in response to untreated and acid and acidic bile salt treated HEMF conditioned media. (A) Fluorescence proliferation assay. Epithelial cells (STR, 600 cells/well of 96 well plate) were treated with conditioned media of untreated HEMFs (cSFMM) or conditioned media from pH 4.5 and pH 4.5 bile salt treated HEMFs, at 1:1, 1:2, 1:4; and 1:8 dilutions in SFMM for 48 hrs. Controls were undiluted keratinocyte serum-free media (KSFM, ThermoFisher Scientific) and serum-free, EGF-free myofibroblast media (SFMM). * p < 0.05 vs. cSFMM. Proliferation was assessed with alamarBlue assay. (B) Luminescence proliferation assay. STR were plated overnight (2000 cells/well of 96 well plate) and then cultured for 96 hours with KSFM, or with SFMM, cSFMM, or conditioned media from HEMFs treated with pH 4.5 or pH 4.5 bile salts, all diluted 1:1 in KSFM, as described in the methods. On day 4, CellTiter-Glo assay reagents was added per manufacturer’s instructions and luminescence read. * p < 0.05 vs. KSFM and SFMM; ** p < 0.05 vs cSFMM. In panel A, ** is p < 0.05 vs cSFMM for the indicated dilution.

To further evaluate the effect of HEMF conditioned media on basal cell proliferation, we utilized 2D culture given that the immortalized human esophageal squamous epithelial cell line grown in 2D culture has a predominantly basal cell phenotype [11, 12]. We evaluated epithelial proliferation with fluorescence and luminescent cell proliferation assays. We first used the alarmarBlue cell viability reagent, which assesses the reducing power of living cells, to quantitatively measure viability. Epithelial proliferation increased 5-fold with conditioned media from untreated HEMFs (cSFMM) compared to unconditioned HEMF media (SFMM), consistent with our prior work [8]. Using undiluted samples, epithelial cells proliferated 1.6-fold and 1.8-fold less in response to conditioned media from pH 4.5 and pH 4.5 bile salt treated HEMFs, respectively, at 48 hours compared to untreated HEMF conditioned media (p<0.05). Dose dilution response was observed with all treatments (Fig 5A). Of note, compared to proliferation with the epithelial cell media, keratinocyte serum free medium (KSFM), proliferation was markedly less with conditioned HEMF media across all treatment conditions. Epithelial cell proliferation was therefore also assessed with a luminescence-based assay, Celltiter-Glo, which determines the number of viable, metabolically active cells in culture based on ATP quantitation (Fig 5B). As described in the methods, pilot studies were conducted to optimize treatment conditions such that epithelial proliferation under control conditions would be similar to that achieved with epithelial cell media. Epithelial cells were cultured with serum-free myofibroblast media (SFMM) or conditioned media from untreated (cSFMM), pH 4.5, or pH 4.5 bile salt treated HEMFs diluted 1:1 with KSFM. Under these conditions epithelial cell proliferation is similar in the presence of KSFM and SFMM. After 96 hours, epithelial cells cultured with pH 4.5 bile salt treated HEMFs proliferated 1.6-fold less than epithelial cells cultured with untreated HEMF conditioned media. There was a non-significant trend in a decrease in proliferation of epithelial cells cultured with conditioned media from pH 4.5 treated HEMFs. Overall, using two different proliferation assays, in the 2D in vitro culture model conditioned media from pH 4.5 and pH 4.5 bile salt treated HEMFs decreased squamous cell basal epithelial proliferation compared to conditioned media from untreated HEMFs.

## Discussion

We have demonstrated for the first-time paracrine regulation of squamous epithelial phenotype by HEMFs stimulated with noxious luminal signals encountered in GERD. Conditioned media from pH 4.5 and pH 4.5 bile salt treated HEMFs increased squamous epithelial thickness in a 3D organotypic-like air-liquid interface (ALI) culture model that allows for investigation of epithelial-HEMF paracrine interactions. In this model, HEMFs are exposed to acid and acidic bile salts in a manner that mimics GERD and then epithelial cells are cultured with HEMF conditioned media in 3D organotypic culture. In humans with gastroesophageal reflux, exposure of the esophageal epithelium occurs on the luminal, stratified epithelial side. Our model is physiologically relevant in that dilated intercellular spaces or erosions present in uncomplicated and complicated GERD allow for penetration of noxious luminal agents through a disrupted epithelial barrier to reach the myofibroblasts in the subepithelial space. Alternatively, given the association between GERD and obesity, which is an inflammatory condition, and esophageal adenocarcinoma, which is also a model of an inflammation associated cancer [13], it is conceivable that stromal cells in the subepithelial space could be exposed directly to inflammatory signals, again providing an opportunity for myofibroblast involvement.

Interestingly, an increase in epithelial thickness has been described as a distinguishing feature of GERD in human esophageal biopsies [1, 2]. In this study, we begin to investigate for the first time the role of esophageal stromal cells in mediating the increase in thickness observed in GERD. We show for the first time that secreted factors from HEMF stimulated with acidic components of GERD (acid, acidic bile salts) mediate an increase in epithelial thickness in a 3D organotypic like-ALI model. This model utilizes HEMF conditioned media and therefore allows us to demonstrate a paracrine mediated mechanism. In this model, bile salts may not be playing a major role given that acidity (pH 4.5) alone seems to be sufficient to increase epithelial thickness.

In our model, quantification of the number of p63 immuno-stained basal showed an increase after normalization to total DAPI+ cells in epithelium established with conditioned media from both pH 4.5 and pH 4.5 bile salt treated HEMFs compared to untreated HEMFs. Furthermore, p63+ basal cells of squamous epithelium established with pH 4.5 or pH 4.5 bile salt treated HEMF conditioned media were multilayered. In contrast, p63 expressing basal cells in squamous epithelium established with untreated HEMF conditioned media were monolayered. Beyond being a basal cell marker, p63 is a transcription factor that regulates differentiation and morphogenesis and is required for the formation of squamous epithelia and maintenance of the basal cell proliferation [10]. Others have shown that p63 is downregulated in epithelial cells in culture directly exposed to acid and bile salts [14]. Of note, in our model, epithelial cells are exposed to neither acid nor acidic bile salts. Rather they are exposed to conditioned media from acid and acidic bile salt treated HEMFs, without any direct exposure to these noxious luminal stimuli. Our work raises the possibility that sub-epithelial cells such as HEMFs may also play a role in p63 regulation by promoting basal cell maintenance at some early point in the GERD injury spectrum.

We also show an increase in basal and supra-basal thickness (quantified based on CK14 and CK4 immunostaining, respectively) in epithelium established with conditioned media from pH 4.5 and pH 4.5 bile salt treated HEMF conditioned media. These findings are consistent with the observed increase in squamous epithelial thickness. Although we see a clear increase in epithelial thickness and an increase in basal cell content in epithelium established with conditioned media from pH 4.5 and pH 4.5 bile salt treated HEMFs, we did not observe an associated increase in proliferation, at least as measured with PCNA. There was no increase in PCNA + cells across treatment conditions when PCNA quantification was normalized to μm of the epithelium. When PCNA quantification was normalized to total DAPI + cells, however, there was a decrease in the PCNA +/DAPI + ratio. This finding suggests that the total number of DAPI+ cells was increased under acidic conditions and that acidic conditions may have increased cell proliferation, at least in the beginning. Such a notion is corroborated by increased p63+ basal cells and the greater number of total DAPI + cells in thicker epithelium established with acid treated HEMFs. The discrepancy between p63 and PCNA immunostaining also suggests that perhaps not all p63+ cells were proliferating.

To further investigate the effect of HEMF secreted factors on basal cell proliferation, we evaluated basal cell proliferation in 2D culture of squamous epithelial cells which reflect the phenotype of undifferentiated cells of the basal layer [11]. Cell viability data from both fluorescence and luminescence based assays demonstrate an increase in epithelial proliferation in the presence of conditioned media from untreated HEMFs compared to unconditioned HEMF media. This finding is consistent with our prior observation of an increase in proliferation in epithelium established with conditioned HEMF media vs either KSFM or unconditioned HEMF media [8]. In both assays, epithelial cell proliferation in the presence of conditioned media from acid treated HEMFs, was less than epithelial proliferation in the presence of conditioned media from untreated HEMFs. Differences in epithelial cell proliferation between the control media (KSFM and SFMM) in the fluorescence and luminescence based assays are accounted by differences in media preparation in the two assays described in detail in the methods. HEMF derived samples in the fluorescence assay were EGF-free while those in the luminescence cell viability assay were diluted 1:1 with KSFM. Regardless, neither of these assays determine cell proliferation directly but rather assess mitochondrial functions to estimate the number of live cells. Therefore, the applicability of cell viability data in monolayer culture to the 3D organotypic model or *in vivo* state may be limited.

While the reasons for this apparent inconsistency between epithelial thickness, basal cell hyperplasia, and epithelial proliferation may not be fully reconciled in this manuscript, work by others in human esophageal epithelial 3D organoid models shows that increases in basal cells may not be accounted for increased proliferation but instead delays in differentiation [12]. Other potential explanations not addressed in this manuscript include the role of different rates of apoptosis in epithelium established with conditioned media from untreated vs treated HEMFs. In addition, evaluation at earlier time points or with an alternate proliferation markers in both models such as Ki67 and BrdU which measure different components of the cell cycle [15, 16] may shed further light on the apparent discrepancy. We suspect that there must be an earlier time point when epithelial cell proliferation is increased, resulting the formation of thicker epithelia and more CK14 +/p63 + basal cell content.

In conclusion, our novel studies demonstrate for the first time paracrine effects of HEMFs stimulated with acidic stimuli on squamous epithelium. As of yet unidentified secreted factors from pH 4.5 and pH 4.5 bile salt treated HEMFs increase epithelial thickness in an 3D OTC-ALI model. These factors partially increase p63 expression and expand basal and supra-basal components of the epithelium without a clear increase in basal cell proliferation. The effects of secreted factors from acid treated HEMFs on basal cell proliferation in this model and the mechanism mediating the increase in epithelial thickness merit further investigation. Studies to explore the signaling pathways mediating this phenotype are ongoing. Our work has implications for improved understanding of GERD pathogenesis, thereby allowing identification of novel therapeutics.

## Supporting information

S1 File Supporting data for Fig 1. (XLSX)

S2 File Supporting data for Fig 2. (XLSX)

S3 File Supporting data for Fig 3. (XLSX)

S4 File Supporting data for Fig 4. (XLSX)

S5 File Supporting data for Fig 5. (XLSX)
